# *Rickettsia* spp. in Ticks of South Luangwa Valley, Eastern Province, Zambia

**DOI:** 10.3390/microorganisms11010167

**Published:** 2023-01-09

**Authors:** Bruno S. J. Phiri, Simone Kattner, Lidia Chitimia-Dobler, Silke Woelfel, Celina Albanus, Gerhard Dobler, Thomas Küpper

**Affiliations:** 1 Central Veterinary Research Institute (CVRI), Ministry of Fisheries and Livestock, Lusaka P.O. Box 33980, Zambia; 2 Institute of Occupational, Social and Environmental Medicine, RWTH Aachen University, Pauwelsstrasse 30, 52074 Aachen, Germany; 3 Bundeswehr Institute of Microbiology, Neuherbergstrasse 11, 80937 Munich, Germany; 4 German Center of Infection Research (DZIF) Partner Munich, 80937 Munich, Germany; 5 Amedes MVZ for Laboratory Medicine and Microbiology, 82256 Fürstenfeldbruck, Germany; 6 Parasitology Unit, University of Hohenheim, 70599 Stuttgart, Germany

**Keywords:** ticks, *Rickettsia* spp., Zambia

## Abstract

Ticks are important vectors for *Rickettsia* spp. belonging to the Spotted Fever Group responsible for causing Rickettsiosis worldwide. Rickettsioses pose an underestimated health risk to tourists and local inhabitants. There is evidence of the presence of *Rickettsia* spp. in Zambia, however there is limited data. A total of 1465 ticks were collected in 20 different locations from dogs and cattle including one cat. Ticks were identified by morphological features or by sequencing of the 16S mitochondrial rRNA gene. Individual ticks were further tested for rickettsiae using a pan-Rickettsia real-time-PCR. *Rickettsia* species in PCR-positive ticks were identified by sequencing the 23S-5S intergenic spacer region or partial ompA gene, respectively. Seven tick species belonging to three different tick genera were found, namely: *Amblyomma variegatum*, *Rhipicephalus appendiculatus*, *Rhipicephalus* (Boophilus) *microplus*, *Rhipicephalus simus*, *Rhipicephalus sanguineus*, *Rhipicephalus zambesiensis* and *Haemaphysalis elliptica*. Out of the 1465 ticks collected, 67 (4.6%) tested positive in the pan-Rickettsia PCR. This study provides detailed data about the presence of *Rickettsia* species in South Luangwa Valley, Eastern Province, Zambia for the first time. High prevalence of *Rickettsia africae* in *Amblyomma variegatum* was found, which indicates the potential risk of infection in the investigated area. Furthermore, to our best knowledge, this is the first time *Rickettsia massiliae*, a human pathogen causing spotted fever, has been detected in Zambia.

## 1. Introduction

Rickettsioses of the Spotted Fever Group (SFG) are common in many sub-Saharan African countries [1,2,3]. Several human pathogenic *Rickettsia* spp. are known to occur in Zambia’s neighboring states [1,3,4]. Although known vectors of rickettsiae such as *Amblyomma (A.) variegatum*, *Rhipicephalus (Boophilus) microplus*, *Rhipicephalus (Rh.) simus*, *Rhipicephalus sanguineus*, *Rhipicephalus zambesiensis* and *Rhipicephalus appendiculatus* occur in Zambia [3,5,6,7,8], the medical importance of rickettsioses in Zambia is unclear and epidemiological data is scarce. So far, only one study has reported IgG antibodies against SFG rickettsiae in Zambia’s inhabitants, thus indicating the occurrence of rickettsial infections in humans [9]. The highest prevalence of SFG *Rickettsia* antibodies was found in Chipata district in Eastern Province of Zambia [9]. In addition, the occurrence of anti-rickettsial antibodies in baboons and vervet monkeys in South and North Luangwa National Park confirmed this observation [10], while more detailed studies on the detection and identification of rickettsial species in Zambia are not available. Another study described *Rickettsia* (R.) *africae* in *Amblyomma sparsum* on tortoises imported to Japan from Zambian tortoise populations [11], as the only direct confirmation of the occurrence of this human pathogen in Zambia.

The aims of this study were to show the prevalence of SFG rickettsiae in ticks and to ascertain which species of SFG rickettsiae occur in South Luangwa Valley. As many SFG rickettsiae are human pathogens, these data are of great importance for travel medicine In addition, the data will add value to the knowledge of local general practitioners concerning the possible causes of febrile diseases in the local population [12].

## 2. Materials and Methods

Ethical clearance to conduct the study was obtained from ERES-CONVERGE IRB in Zambia (Ref. No. 2022-July-001).

### 2.1. Sampled Area and Tick Collection

**Figure 1:**
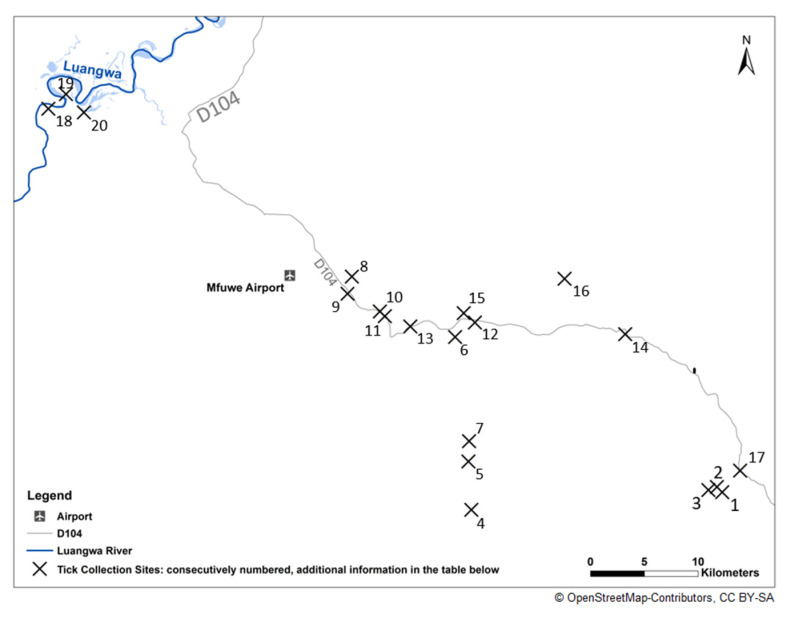
Map of locations of tick collection. Legend: Kakumbi: No. 18, 19, 20; Chikowa: No. 5, 7; Jumbe: No. 8–13, 15, 16; Mphomwa: No. 1–4. 6, 14, 17; The map was designed in ArcGIS 10.2.2 (Copyright © 1995–2014 Esri) using open source data of ©OpenStreetMap-Contributors (license CC BY-SA).

Ticks were collected in the chiefdoms Jumbe and Kakumbi in the Mambwe district, South Luangwa Valley, of Zambia’s Eastern Province. Tick sample collection and laboratory analysis were performed between 19 July 2022 and 16 September 2022 (Figure 1). Collection sites were located between 500 and 800 m above sea level and spread over an area of approximately 2093 km^2^. Ticks were collected from watchdogs, cattle and one pet cat. The ticks were stored at 4 °C, transported to Germany alive and stored at −80 °C until identification and processing. The map with the collection sites was designed in ArcGIS 10.2.2 (Copyright © 1995–2014 Esri) using open source data of ©OpenStreetMap-Contributors (license CC BY-SA, [13]).

### 2.2. Collection Sites

Ticks were collected from dogs and cattle of small farms located individually or in little villages in the chiefdom Jumbe’s wards Mphomwa, Chikowa and Jumbe (Figure 1). These farms kept pigeons, chicken and grow crops. In the eastern part of Mphomwa ward, farms were breeding cattle (Location 3, Figure 1). Farmland and natural vegetation surrounded the study site. The cattle were grazing free-ranged during day and were fenced at night.

In addition, some samples were collected from a cat and two dogs in homes with well-tended gardens at the riverside of the Luangwa in Kakumbi chiefdom in Kakumbi ward (Table 2, Figure 1). Largely, the original vegetation around the houses and gardens remained unchanged. Due to the direct proximity of the South Luangwa National Park across the river there was no land or livestock farming in this region. Different antelope species and other wildlife species were observed regularly in the immediate vicinity of the houses.

### 2.3. Identification of Tick Species

The ticks were identified using morphological characters according to Walker et al. [14,15] and Apanaskevich et al. [16]. For ticks, which could not be unambiguously identified by morphology, molecular identification was performed using a polymerase chain reaction (PCR) protocol targeting the mitochondrial 16S rRNA gene as described by Halos et al. [17].

### 2.4. Tick Preparation and Nucleic Acid Extraction

After identification, 1051 ticks were homogenized individually, 414 ticks in pools up to 7 individuals in 800 µL minimal essential medium (MEM, Life Technologies), using Lysing matrix tubes A (MP Biomedicals) in a FastPrep Instrument BIO101 (MP Biomedicals). Total nucleic acid was extracted from 200 µL homogenate using the MagNA Pure LC RNA/DNA Kit (Roche, Mannheim, Germany) in a MagNA Pure LC instrument (Roche), according to the manufacturer’s instructions. The total nucleic acid was stored at −80 °C until use.

### 2.5. Detection of Rickettsia spp. in Tick Samples

The ticks were tested for *Rickettsia* spp. using a pan-Rickettsia real-time-PCR, targeting the citrate synthase gene [18]. All specimens with a CT-value above zero were rated as positive and processed in molecular identification. The molecular identification to species level was achieved by analyzing the ompA fragment IV and the 23S-5S intergenic spacer region according to previously published protocols [19,20]. (Table 1).

### 2.6. Sequence Analysis and Phylogenetic Classification

Obtained PCR products were purified using the QIAquick^®^ PCR Purification Kit 205 according to the manufacturer’s instructions. For identification, the amplicons were sequenced using Sanger technique by an external contractor (GATC Biotech, Konstanz, Germany). Sequences were analyzed using BioEdit Alignment Editor (Version 7.2.5.0) [21] and compared with the gene bank of NCBI (National Centre for Biotechnology Information) [22] by BLAST (Basic Local Alignment Search Tool) [23]. Phylogenetic analysis was performed in MEGA 7 [24]. For multiple sequence alignment Clustal W in MEGA 7 was used [25]. Molecular phylogenetic analysis was performed by the Maximum Likelihood method using the Tamura-Nei model [26] and bootstrap method (1000 replications). A maximum likelihood phylogenetic tree was created for the partial gene sequences of 23S-5S rRNA and ompA fragment IV. Identical clusters and similar confidence values were obtained by the Maximum Parsimony method and the Neighbour-joining method.

### 2.7. Data Analysis

To perform data analysis, chi^2^-test and adjusted Pearson’s coefficient of contingency were conducted in Microsoft^®^ Excel 2016. For all chi^2^-test analysis a *p*-value of < 0.05 was defined as statistically significant. For Pearson’s coefficient of contingency values between 0 to 0.2 were seen as week association, between 0.2 to 0.6 average and higher than 0.6 as high association between the tested characteristics.

## 3. Results

A total of 1465 ticks were collected at 20 different locations spread over 4 wards and from different hosts: 1438 from dogs (98.2%), 24 ticks from cattle (1.6%), and three ticks from one cat (0.2%). The ticks belonged to three genera and seven species. Percentage of species distribution, prevalence of *Rickettsia* spp. and infection rates are listed in Table 2.

In the wards Chikowa and Jumbe, only dogs were sampled. In Chikowa, none of the 40 collected ticks (8 *Rh. appendiculatus*, 32 *Rh. sanguineus* “tropical lineage” [27]) were positive in pan-Rickettsia real-time-PCR. In Jumbe, one of 764 (0.1%) specimens was positive in pan-Rickettsia PCR and was identified as *R. conorii* ssp. *caspia* [28]. In detail 5 *Rh. zambesiensis*, 7 *Rhipicephalus simus*, 26 *H. elliptica*, 99 *Rh. appendiculatus*, 626 *Rh. sanguineus* “tropical lineage” were negative for *Rickettsia* spp. and one *Rh. sanguineus* “tropical lineage” was positive (cf. Table 3).

In ward Mphomwa, 66 of 656 (10.1%) collected ticks were found positive in pan-Rickettsia real-time-PCR (cf. Table 3). Of these 66 positive ticks, 40 *Rh. sanguineus* “tropical lineage” and four *A. variegatum* were collected from dogs and another 21 *A. variegatum* and one *Rh. (B.) microplus* were collected from cattle. From the 66 positive ticks, 27 were identified as *R. africae*: 20 *A. variegatum* collected from cattle, four *A. variegatum* and three *Rh. sanguineus* “tropical lineage” collected from dogs. One *Rh. sanguineus* “tropical lineage” (1/594) collected from dogs was positive for *R. massiliae*. For the remaining 38 *Rickettsia*-positive specimens: 36 *Rh. sanguineus* “tropical lineage” were collected from dogs and one *Rh. (B.) microplus* and one *A. variegatum* were collected from cattle no further determination by sequencing was possible, due to low amount of DNA of these samples. (Table 3)

In Kakumbi, neither the two *A. variegatum* and the one *Rh. sanguineus* “tropical lineage” collected from a cat nor the two females (*Rh. sanguineus* “tropical lineage” and *Rh. appendiculatus*) from two dogs were positive for *Rickettsia* spp. (Table 3).

From the total 1255 collected *Rh. sanguineus* “tropical lineage” specimens, for *R. massiliae* and *R. conorii* ssp. *caspia* [28] a prevalence of 0.08%, respectively, was calculated. The prevalence of *R. africae* in *Rh. sanguineus* “tropical lineage” was found to be 0.24% with a median CT-value of 35, 28 in the pan-Rickettsia real-time-PCR. From 30 *A. variegatum* specimens, 24 *A. variegatum* (80%) were found positive with a median CT-value of 26,71 for *R. africae* (Table 2 and Table 3).

### 3.1. Phylogenetic Analysis

**Figure 2:**
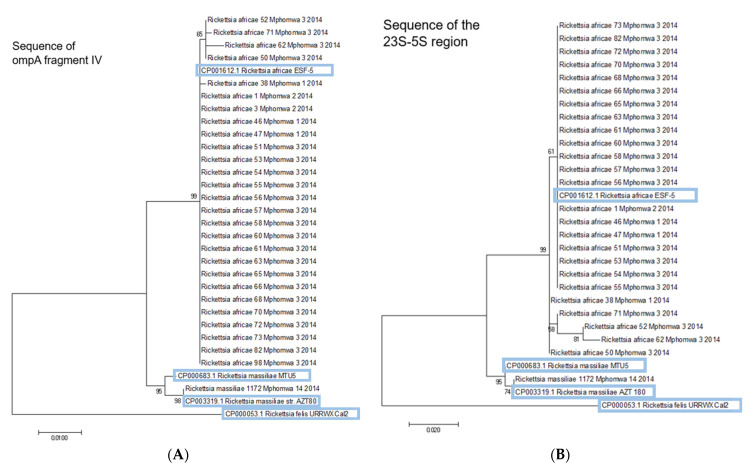
Phylogenetic relationship between *Rickettsia* spp. by (**A**) ompA gene and (**B**) 23S-5S intergenic spacer region. Legend Figure 2: The number at the branches gives the percentage of trees in which these sequences are clustered together. The bar indicates the percentage of sequence divergence. Label: *Rickettsia* species | Sample No. | location No (cf. Figure 1) | Date of collection.

The majority of detected *R. africae* showed 100% identity in both analyzed genomic targets to the type strain gb|CP001612.1 *R. africae* ESF-5. In the ompA target the phylogenetic analysis revealed one additional, well-supported clade of slightly different *R. africae* variants with identities from 99.4–99.9% to the type strain *R. africae* ESF-5 (Figure 2A,B). The phylogenetic investigation of the hypervariable 23S-5S intergenic spacer region showed a stronger variability, resulting in three additional well supported clades with identities from 98.3–99.7% to the type strain *R. africae* ESF-5 (Figure 2A,B). The repeated analysis of the 23S-5S intergenic spacer region of sample 3, at location 2 (Figure 1), revealed no known sequence of a *Rickettsia* species but the sequence analysis of partial ompA revealed a 100% identity to *R. africae* ESF-5. The *R. massiliae* sequence of sample 1172 was 100% identical to gb|CP003319.1 *R. massiliae* str. AZT80 in both investigated genomic targets, respectively. Sample 582 showed the closest phylogenetic relationship to gb|AH011786.2 *R. conorii* ssp. *caspia* Chad (partial ompA 100%) and gb|U83437.1 *R. conorii* ssp. *caspia* A-167 (23S-5S intergenic spacer 100%) [28].

### 3.2. Statistical Analysis

The statistical analysis by chi^2^-test and adjusted Pearson’s coefficient of contingency (P) revealed that the spread of tick species was strongly dependent on the host (Table 4). Additionally, the positive or negative CT-value was strongly dependent on the tick species and the host of the tick. There was a low to middle association between the positive or negative CT-value and the ward where the specimens were collected. There was also a low to middle association between ward and the tick species.

## 4. Discussion

Although *R. africae* is probably the most common rickettsial species in Africa, surprisingly only few studies are available to test genetic differences in isolates or strains of *R. africae* from different locations. In our study, the analysis of the partial ompA gene showed two and the 23S-5S intergenic spacer region revealed four different genetic clusters, which occurred in one single location named Mphomwa 3 (cf. Figure 1 and Figure 2). The 23S-5S intergenic spacer region is more suitable to detect minor genetic changes due to its hypervariability. However, the reliability of these changes is lower because mutations in non-coding regions producing distinct changes will usually not cause any phenotypic changes in the bacterial organism and therefore might accumulate randomly. It has to be studied in more detail whether the observed genetic differences result in phenotypic differences, e.g., regarding pathogenicity and if they provide more insight in the evolution and dispersal of *R. africae* on the African continent.

*Rickettsia africae* is one of the most widespread rickettsial species in Africa [2,29]. Therefore, it was expected to find a high prevalence of *R. africae* in *A. variegatum* ticks, as it has been observed in other African countries [3]. Three *Rh. sanguineus* “tropical lineage” ticks were positive for *R. africae*. In this tick species *R. africae* is rarely found and might be only a contaminant from rickettsiaemic blood of the host. This assumption was supported by the fact that the amount of rickettsial DNA in *Rh. sanguineus* “tropical lineage” was low in comparison to *A. variegatum*. The statistical analysis also showed a strong association between the positive CT-value and *A. variegatum*, respectively, the negative CT-value and *Rh. sanguineus* “tropical lineage”. In line with our own data several other studies found that the prevalence of *R. africae* in ticks from the genus *Rhipicephalus* is always lower than in the genus *Amblyomma* [30,31].

In the present study, *R. africae* was reported only in the eastern part of Mphomwa ward (Locations 1, 2, 3 in Figure 1). A likely reason for this focal occurrence is that exclusively in this region, ticks collected from cattle were obtained, being represented solely by *A. variegatum* with a high prevalence for *R. africae*. The Pearson’s coefficient of contingency showed a strong association between the host and the tick species which is in line with literature saying *A. variegatum* is mostly found on cattle and *Rh. sanguineus* mostly on dogs. Usually, such a focal distribution of *R. africae* is unusual as this species is generally found endemically over large areas in Africa. High prevalence rates of *R. africae* in *Amblyomma* ticks are seen in pasture settings in different countries in Africa [3]. The host animal at the location is a basic characteristic of the location as it is influenced by man and not by a natural distribution. Therefore, it is not possible to test an association by Pearson for host and location. As Maina et al. suggested, cattle are not the reservoir host of *R. africae* [32]. However, in this study a strong association between cattle, *A. variegatum* and *R. africae* was found. If looking at the data of ticks from dogs only (Table 4), there is a low to middle association between the ward and a positive or a negative specimen. Furthermore, there is a low to middle association of the place of collection and the tick species. Accordingly, there have to be further important environmental factors affecting tick abundance and by that the positivity for rickettsiae aside from the host. It is assumed that small mammals, large antelopes, and ungulates may serve as vertebrate hosts. This relation concurs with knowledge in travel medicine that certain behavioral factors, such as game hunting and excessive contact to ground vegetation as well as environmental factors and travelling during rainy season and high humidity, increase the risk of an infection with *R. africae* in tourists [33]. Additionally, the natural transmission cycle of *R. africae* has not been elucidated, so far. Moreover, it is known that transovarial transmission and transstadial persistence may play a major role for the maintenance of rickettsiae in the vectors while the role of vertebrates is still not completely understood [34]. Therefore, our results may implicate that one or more essential factors for the natural transmission cycle of *R. africae* are present in the eastern region of the study area but seemingly not in other regions. However, these essential factors have yet to be identified.

*Rickettsia africae* is the etiologic agent of African tick-bite fever. This is usually a benign febrile disease without any complications or sequelae. Human infection might cause malaria-like symptoms, and therefore, could be under- or misdiagnosed.

In this study, *R. massiliae* was detected for the first time in ticks in Zambia and also in its Eastern Province. This rickettsial species is known to occur in Africa [1,3,30]. A study by Cicuttin et al. suggested the prevalence of *R. massiliae* in the genus *Rhipicephalus* to be 5–6% in North Africa [35]. Although it has been detected mainly in northern and eastern Africa, so far, *R. massiliae* was also recently described in South Africa [36] and in Botswana [31]. Therefore, the detection in Zambia in the tick species *Rh. sanguineus* “tropical lineage” is in line with published data [1,3,4,35,37]. Our data indicate a very low prevalence of *R. massiliae* in Zambia as it could be detected only in one *Rh. sanguineus* “tropical lineage” in the ward Mphomwa (Location 14 in Figure 1). Recently, it was shown that at least three genetic forms of *Rh. sanguineus* s. l. occur in different parts of the world, which exhibit different patterns of vector competence [27,38]. It is assumed that *R. massiliae* has its main vertebrate host in dogs and canids [35]. The low prevalence rate in *Rh. sanguineus* “tropical lineage” might indicate, that the circulating genetic form of *Rh. sanguineus* “tropical lineage” might be an incompetent vector for the local *R. massiliae* strain and therefore occur only in a very low frequency in this vector. It is known that *R. massiliae* is not common in rural areas [4]. Therefore, it has to be studied whether it can be found more regularly in urbanized areas in Zambia. *Rickettsia massiliae* was reported to cause human diseases comparable with Mediterranean spotted fever [39]. In Argentina, it was the agent of a severe form of spotted fever [37]. So far, no clinical forms of human *R. massiliae* infections have been described in Africa, but it may be the cause of undifferentiated febrile diseases in the region that, due to the lack of knowledge, remain unrecognized.

Finally, *R. conorii* ssp. *caspia* was detected in a single sample [28]. So far, only one other study has reported the occurrence of this *Rickettsia* ssp. much further north in Chad [40]. As *R. conorii* ssp. *caspia* has been described as causative agent of Astrakhan fever with the possibility of hemorrhagic manifestation in Russia [41] it must be taken into consideration as a possible cause of hemorrhagic fever manifestations in Zambia and the whole southern African region.

## 5. Conclusions

We studied a tick population collected from dogs, cattle and one cat in Zambia. In total, seven different tick species were detected. In *A. variegatum* and *Rh. sanguineus* “tropical lineage” three pathogenic rickettsiae, namely *R. africae*, *R. massiliae* and *R. conorii* ssp. *caspia* were detected. The prevalence of *R. africae* was localized and occurred only in an agricultural area with cattle breeding. The impact of the observed genetic variability of *R. africae* on pathogenic features has to be clarified in future studies. A second rickettsial species, *R. massiliae*, was found in one *Rh. sanguineus* “tropical lineage” for the first time in Zambia.

## Figures and Tables

**Table 1:** Primers and Probes used for molecular investigation in this study.

Target (Partial)	Forward Primer Reverse Primer (Probe)	Product Size	References
16S rRNA	TQ16S+F1 (5′-CTGCTCAATGATTTTTTAAATTGCTGTGG-3′) TQ-16S-2R (5′-ACGCTGTTATCCCTAGAG-3′)	320 bp	[17]
*glt*A real-time	PanRick_gltA_2_for: (5′-ATAGGACAACCGTTTATTT-3′) PanRick_gltA_2_rev: (5′-CAAACATCATATGCAGAAA-3′) PanRick_3_taq: (5′-6FAM-CCTGATAATTCGTTAGATTTTACCG-TMR-3′)	70 bp	[18]
*omp*A IV	RR-5125 (5′-gCggTTACTTTAgCCAAAgG-3′) cRR-6013: (5′-TCTTCTgCgTTgCATTACCg-3′)	888 bp	[19]
23S-5S intergenic spacer	Rick 23s for (5′- GATAGGTCGGGTGTGGAAGCAC) Rick 23s rev (5′- GGGATGGGATCGTGTGTTTCAC)	300–550 bp (species-dependent)	[20]

**Table 2:** Total number of different tick species and *Rickettsia* positive specimens per tick species.

Tick Species	Ticks n (%)	Rickettsia Positive n (Prevalence in Tick Species)	SE	95% CI [Lower Limit; Upper Limit]
*Amblyomma variegatum*	30 (2.1)	25 (0.833)	0.068	[0.68; 0.95]
*Haemaphysalis elliptica*	29 (2.0)	0 (0)	0	
*Rhipicephalus appendiculatus*	137 (9.4)	1 (0.007)	0.007	[0; 0.02]
*Rhipicephalus (Boophilus) microplus*	1 (0.1)	1 (1)	0	n.a.
*Rhipicephalus sanguineus* “tropical lineage”	1254 (85.6)	40 (0.032)	0.005	[0.02; 0.04]
*Rhipicephalus simus*	9 (0.6)	0 (0)	0	
*Rhipicephalus zambesiensis*	5 (0.3)	0 (0)	0	
**Total**	**1465**	**67 (0.046)**	**0.005**	**[0.03; 0.06]**

Legend: SE (Standard Error), CI (Confidence Interval), n.a. (not applicable).

**Table 3:** List of specimens with collection site, host, tick species, stage and the result of pan-Rickettsia PCR.

Chiefdom, Ward	Host Species	Tick Species (% of Total Number of the Ward)	Stage	Total *Rickettsia* Positive/Total Tested (%)
Jumbe, Chikowa	Dog	*Rh. appendiculatus* (20%)	f	0/3 (0)
			m	0/5 (0)
		*Rh. sanguineus* “tropical lineage” (80%)	f	0/11 (0)
			m	0/21 (0)
		Total Chikowa		0/40 (0)
Jumbe, Jumbe	Dog	*H. elliptica* (3.4%)	f	0/16 (0)
			m	0/10 (0)
		*Rh. appendiculatus* (13%)	f	0/53 (0)
			m	0/46 (0)
		*Rh. sanguineus* “tropical lineage” (82%)	f	0/256 (0)
			m	1/340 (0.3)
			n	0/31 (0)
		*Rh. simus* (0.9%)	f	0/3 (0)
			m	0/4 (0)
		*Rh. zambesiensis* (0.7%)	f	0/2 (0)
			m	0/3 (0)
		Total Jumbe		1/764 (0.13)
Jumbe, Mphomwa	Dog	*A. variegatum* (0.8%)	f	3/3 (100)
			m	1/1 (100)
			n	0/1 (0)
		*H. elliptica* (0.5%)	f	0/1 (0)
			m	0/1 (0)
			n	0/1 (0)
		*Rh. appendiculatus* (4.3%)	f	0/10 (0)
			m	0/18 (0)
		*Rh. sanguineus* “tropical lineage” (90%)	f	20/241 (8.3)
			m	20/352 (5.7)
			n	0/1 (0)
		*Rh. simus* (0.3%)	m	0/2 (0)
		Total Dog		44/632 (7.0)
	Cattle	*A. variegatum* (3.5%)	f	21/23 (91.3)
		*Rh. (B.) microplus* (0.2%)	f	1/1 (100)
		Total Cattle		22/24 (91.7)
		Total Mphomwa		66/656 (10.1)
Kakumbi, Kakumbi	Dog	*Rh. appendiculatus* (20%)	f	0/1 (0)
		*Rh. sanguineus* “tropical lineage” (20%)	f	0/1 (0)
		Total Dog		0/2 (0)
	Cat	*A. variegatum* (40%)	f	0/2 (0)
		*Rh. sanguineus* “tropical lineage” (20%)	f	0/1 (0)
		Total Cat		0/3 (0)
		Total Kakumbi		0/5 (0)
Total				67/1465 (4.6)

Legend 2: f = female, m = male, n = nymph.

**Table 4:** Results of the Chi^2^ test and the adjusted Pearson’s coefficient of contingency (P).

	Degrees of Freedom	Chi^2^ Critical Value	Chi^2^ Value	P	Result
Tick species and C_T_-value	6	12.9	460.79	0.7	strong association
Tick species* and C_T_-value	3	9.49	441.24	0.7	
Host and C_T_-value	2	5.99	424.15	0.7	Strong association
Host* and C_T_-value	1	3.84	423.18	0.7	
Ward and C_T_-value	3	7.81	81.97	0.3	low to middle association
Ward_+_ and C_T_-value	3	7.81	53.56	0.3	
Host and tick species	12	21.03	1197.65	0.8	strong association
Host* and tick species*	3	7.81	1147.48	0.9	
Ward and tick species	18	28.87	166.83	0.4	middle association
Ward_+_ and tick species_+_	15	25	104.33	0.3	low to middle association

Legend 4: The level of significance was in all test 5% (*p*-value = 0,05). Legend: tests without small sample amounts labeled with *: tick species* without *Rh. (B.) microplus*, *Rh. zambesiensis*, *Rh. simus*; tick species_+_ only from dogs; host* without samples of the cat; ward_+_ only samples of dogs.

## Data Availability

Data was obtained through the Central Veterinary Research Institute (CVRI), Ministry of Fisheries and Livestock, Department of Veterinary Services, Zambia and the Department of Microbiology of the German Armed Forces and are available from the authors with the permission of the providing parties.

## References

[1] Mediannikov O., Diatta G., Fenollar F., Sokhna C., Trape J.F., Raoult D. (2010). Tick-borne rickettsioses, neglected emerging diseases in rural Senegal. PLoS Negl. Trop. Dis..

[2] Mtshali K., Khumalo Z., Nakao R., Grab D.J., Sugimoto C., Thekisoe O. (2016). Molecular detection of zoonotic tick-borne pathogens from ticks collected from ruminants in four South African provinces. J. Vet. Med. Sci..

[3] Parola P., Paddock C.D., Socolovschi C., Labruna M.B., Mediannikov O., Kernif T., Abdad M.Y., Stenos J., Bitam I., Fournier P.E. (2013). Update on Tick-Borne Rickettsioses around the World: A Geographic Approach. Clin. Microbiol. Rev..

[4] Heinrich N., Dill T., Dobler G., Clowes P., Kroidl I., Starke M., Ntinginya N.E., Maboko L., Löscher T., Hoelscher M. (2015). High Seroprevalence for Spotted Fever Group Rickettsiae, Is Associated with Higher Temperatures and Rural Environment in Mbeya Region, Southwestern Tanzania. PLoS Negl. Trop. Dis..

[5] Zieger U., Horak I.G., Cauldwell A.E., Uys A.C. (1998). Ixodid tick infestations of wild birds and mammals on a game ranch in central province, Zambia. Onderstepoort J. Vet. Res..

[6] Berkvens D.L., Geysen D.M., Chaka G., Madder M., Brandt J.R. (1998). A survey of the ixodid ticks parasitising cattle in the Eastern province of Zambia. Med. Vet. Entomol..

[7] Makala L.H., Mangani P., Fujisaki K., Nagasawa H. (2003). The current status of major tick borne diseases in Zambia. Vet. Res..

[8] Pegram R.G., Perry B.D., Musisi F.L., Mwanaumo B. (1986). Ecology and phenology of ticks in Zambia: Seasonal dynamics on cattle. Exp. Appl. Acarol..

[9] Okabayashi T., Hasebe F., Samui K.L., Mweene A.S., Pandey S.G., Yanase T., Muramatsu Y., Ueno H., Morita C. (1999). Short report: Prevalence of antibodies against spotted fever, murine typhus, and Q fever rickettsiae in humans living in Zambia. Am. J. Trop. Med. Hyg..

[10] Nakayima J., Hayashida K., Nakao R., Ishii A., Ogawa H., Nakamura I., Moonga L., Hang’ombe B.M., Mweene A.S., Thomas Y. (2014). Detection and characterization of zoonotic pathogens of free-ranging non-human primates from Zambia. Parasit. Vectors.

[11] Andoh M., Sakata A., Takano A., Kawabata H., Fujita H., Une Y., Goka K., Kishimoto T., Ando S. (2015). Detection of *Rickettsia* and *Ehrlichia* spp. in Ticks Associated with Exotic Reptiles and Amphibians Imported into Japan. PLoS ONE.

[12] Dobler G., Wolfel R. (2009). Typhus and other rickettsioses: Emerging infections in Germany. Dtsch. Arztebl. Int..

[13] ©OpenStreetMap-Contributors. www.openstreetmap.org/copyright.

[14] Walker A.R., Bouattour A. (2003). Ticks of domestic animals in Africa: A guide to identification of species.

[15] Walker J.B., Keirans J.E., Horak I.G. (2000). The Genus Rhipicephalus (Acari, Ixodidae): A Guide to the Brown Ticks of the World.

[16] Apanaskevich D.A., Horak I.G., Camicas J.L. (2007). Redescription of Haemaphysalis (Rhipistoma) elliptica (Koch, 1844), an old taxon of the Haemaphysalis (Rhipistoma) leachi group from East and southern Africa, and of Haemaphysalis (Rhipistoma) leachi (Audouin, 1826) (Ixodida, Ixodidae). Onderstepoort J. Vet. Res..

[17] Halos L., Jamal T., Vial L., Maillard R., Suau A., Le Menach A., Boulouis H.J., Vayssier-Taussat M. (2004). Determination of an efficient and reliable method for DNA extraction from ticks. Vet. Res..

[18] Wölfel R., Essbauer S., Dobler G. (2008). Diagnostics of tick-borne rickettsioses in Germany: A modern concept for a neglected disease. Int. J. Med. Microbiol..

[19] Fournier P.E., Roux V., Raoult D. (1998). Phylogenetic analysis of spotted fever group rickettsiae by study of the outer surface protein rOmpA. Int. J. Syst. Bacteriol..

[20] Chitimia-Dobler L., Riess R., Kahl O., Wolfel S., Dobler G., Nava S., Estrada-Pena A. (2017). Ixodes inopinatus-Occurring also outside the Mediterranean region. Ticks Tick-Borne Dis..

[21] Hall T.A. (1999). BioEdit: A user-friendly biological sequence alignment editor and analysis program for Windows 95/98. Nucleic Acids Symp. Ser..

[22] NCBI Resource Coordinators (2016). Database resources of the National Center for Biotechnology Information. Nucleic Acids Res..

[23] Altschul S.F., Gish W., Miller W., Myers E.W., Lipman D.J. (1990). Basic local alignment search tool. J. Mol. Biol..

[24] Kumar S., Stecher G., Tamura K. (2016). MEGA7: Molecular Evolutionary Genetics Analysis Version 7.0 for Bigger Datasets. Mol. Biol. Evol..

[25] Thompson J.D., Higgins D.G., Gibson T.J. (1994). CLUSTAL W: Improving the sensitivity of progressive multiple sequence alignment through sequence weighting, position-specific gap penalties and weight matrix choice. Nucleic Acids Res..

[26] Tamura K., Nei M. (1993). Estimation of the number of nucleotide substitutions in the control region of mitochondrial DNA in humans and chimpanzees. Mol. Biol. Evol..

[27] Chitimia-Dobler L., Langguth J., Pfeffer M., Kattner S., Kupper T., Friese D., Dobler G., Guglielmone A.A., Nava S. (2017). Genetic analysis of Rhipicephalus sanguineus sensu lato ticks parasites of dogs in Africa north of the Sahara based on mitochondrial DNA sequences. Vet. Parasitol..

[28] Chitimia-Dobler L., Dobler G., Schaper S., Kupper T., Kattner S., Wolfel S. (2017). First detection of Rickettsia conorii ssp. caspia in Rhipicephalus sanguineus in Zambia. Parasitol. Res..

[29] Ndip L.M., Biswas H.H., Nfonsam L.E., LeBreton M., Ndip R.N., Bissong M.A., Mpoudi-Ngole E., Djoko C., Tamoufe U., Prosser A.T. (2011). Risk factors for African tick-bite fever in rural central Africa. Am. J. Trop. Med. Hyg..

[30] Mediannikov O., Diatta G., Zolia Y., Balde M.C., Kohar H., Trape J.F., Raoult D. (2012). Tick-borne rickettsiae in Guinea and Liberia. Ticks Tick-Borne Dis..

[31] Portillo A., Perez-Martinez L., Santibanez S., Blanco J.R., Ibarra V., Oteo J.A. (2007). Detection of Rickettsia africae in Rhipicephalus (Boophilus) decoloratus ticks from the Republic of Botswana, South Africa. Am. J. Trop. Med. Hyg..

[32] Maina A.N., Jiang J., Omulo S.A., Cutler S.J., Ade F., Ogola E., Feikin D.R., Njenga M.K., Cleaveland S., Mpoke S. (2014). High prevalence of Rickettsia africae variants in Amblyomma variegatum ticks from domestic mammals in rural western Kenya: Implications for human health. Vector Borne Zoonotic Dis..

[33] Jensenius M., Fournier P.E., Vene S., Hoel T., Hasle G., Henriksen A.Z., Hellum K.B., Raoult D., Myrvang B. (2003). African tick bite fever in travelers to rural sub-Equatorial Africa. Clin. Infect. Dis..

[34] Eremeeva M.E., Dasch G.A. (2015). Challenges posed by tick-borne rickettsiae: Eco-epidemiology and public health implications. Front. Public Health.

[35] Cicuttin G.L., Brambati D.F., Rodriguez Eugui J.I., Lebrero C.G., De Salvo M.N., Beltran F.J., Gury Dohmen F.E., Jado I., Anda P. (2014). Molecular characterization of Rickettsia massiliae and Anaplasma platys infecting Rhipicephalus sanguineus ticks and domestic dogs, Buenos Aires (Argentina). Ticks Tick-Borne Dis..

[36] Halajian A., Palomar A.M., Portillo A., Heyne H., Luus-Powell W.J., Oteo J.A. (2016). Investigation of Rickettsia, Coxiella burnetii and Bartonella in ticks from animals in South Africa. Ticks Tick-Borne Dis..

[37] Garcia-Garcia J.C., Portillo A., Nunez M.J., Santibanez S., Castro B., Oteo J.A. (2010). A patient from Argentina infected with Rickettsia massiliae. Am. J. Trop. Med. Hyg..

[38] Nava S., Estrada-Pena A., Petney T., Beati L., Labruna M.B., Szabo M.P., Venzal J.M., Mastropaolo M., Mangold A.J., Guglielmone A.A. (2015). The taxonomic status of *Rhipicephalus sanguineus* (Latreille, 1806). Vet. Parasitol..

[39] Vitale G., Mansueto S., Rolain J.-M., Raoult D. (2006). Rickettsia massiliae Human Isolation. Emerg. Infect. Dis..

[40] Fournier P.-E., Xeridat B., Raoult D. (2003). Isolation of a Rickettsia Related to Astrakhan Fever Rickettsia from a Patient in Chad. Ann. N. Y. Acad. Sci..

[41] Maleev V.V., Galimzianov Kh M., Lazareva E.N., Poliakova A.M., Astrina O.S., Kudriavtsev V.A., Arshba T.E. (2009). Hemostatic disorders and their implication in the pathogenesis of Astrakhan rickettsial fever. Ter. Arkhiv.

